# ImmtorLig_DB: repertoire of virtually screened small molecules against immune receptors to bolster host immunity

**DOI:** 10.1038/s41598-018-36179-5

**Published:** 2019-02-28

**Authors:** Deepyan Chatterjee, Gurkirat Kaur, Shilpa Muradia, Balvinder Singh, Javed N. Agrewala

**Affiliations:** 1 CSIR-Institute of Microbial Technology, Chandigarh, 160036 India; 2 Indian Institute of Technology Ropar, Rupnagar, 140001 India

## Abstract

Host directed therapies to boost immunity against infection are gaining considerable impetus following the observation that use of antibiotics has become a continuous source for the emergence of drug resistant strains of pathogens. Receptors expressed by the cells of immune system play a cardinal role in initiating sequence of events necessary to ameliorate many morbid conditions. Although, ligands for the immune receptors are available; but their use is limited due to complex structure, synthesis and cost-effectiveness. Virtual screening (VS) is an integral part of chemoinformatics and computer-aided drug design (CADD) and aims to streamline the process of drug discovery. ImmtorLig_DB is a repertoire of 5000 novel small molecules, screened from ZINC database and ranked using structure based virtual screening (SBVS) against 25 immune receptors which play a pivotal role in defending and initiating the activation of immune system. Consequently, in the current study, small molecules were screened by docking on the essential domains present on the receptors expressed by cells of immune system. The screened molecules exhibited efficacious binding to immune receptors, and indicated a possibility of discovering novel small molecules. Other features of ImmtorLig_DB include information about availability, clustering analysis, and estimation of absorption, distribution, metabolism, and excretion (ADME) properties of the screened small molecules. Structural comparisons indicate that predicted small molecules may be considered novel. Further, this repertoire is available via a searchable graphical user interface (GUI) through http://bioinfo.imtech.res.in/bvs/immtor/.

## Introduction

Discovering small molecules that are pharmacologically active due to their ability to allosterically modulate the biological function of a protein, qualify the definition of ‘lead compounds’. Further, some of these small molecules possessing desirable characteristics of stability, solubility, effective functional groups, non-toxic and devoid of any undesirable side effects are successfully termed as molecules that have ‘drug like’ properties^1^. Among the major challenges of drug discovery is the identification of small molecules that satisfy the above criteria. Advancements in chemoinformatics and Computer-Aided Drug Designing (CADD) have revolutionized the process of drug discovery into a fast, cost effective, and reliable approach. Further, such approaches are reasonably much more efficient in terms of screening of small molecules that can act as lead compounds against biological targets^2,3^. An integral part of this computer aided method is the origin of algorithmic approach termed as ‘Virtual Screening’ (VS) that dates back to years of 1970, but has become popular in the late 1990’s^4,5^. Such techniques for identifying pharmacologically active molecules have further gained thrust with the emergence of high throughput, freely available, user-friendly docking software and databases, and the evolution of *in silico* approaches^6,7^. The algorithmic approach of virtual screening can be subdivided into two strategies; Ligand-Based Virtual Screening (LBVS) and Structure-Based Virtual Screening (SBVS)^8–10^. During LBVS process, pharmacophore mapping is employed on molecules that are known to bind to biological targets for identifying potentially novel pharmacophore hits, using similarity searching approach. Such chemical similarity search in terms of identifying molecules with akin shape and configuration is performed against a database^11,12^. On the other hand, SBVS encompasses a modeling approach, wherein binding interactions via protein ligand docking of small molecules, housed in a particular database is performed on its biological target (receptor protein)^13^. Both the approaches are followed up using ranking algorithms that employ scoring functions to shortlist potential ligands, and defining their affinity for its receptor site^14^.

Traditionally, G-Protein-Coupled Receptors (GPCRs) have been the target for identifying small molecules using combination of high throughput and virtual screening approaches^15^. Such strategies have been successful in identifying novel compounds or reducing the side effects of drugs by modifying the existing scaffold^16,17^. Interestingly, various methods including computational approaches have been used in identifying novel small molecules that target immune receptors, like pattern recognition receptors (PRRs)^18–21^, intracellular adhesion molecules^22–24^, and cytokines^25–28^. Relatively economical and high-speed algorithmic approaches like SBVS can screen millions of small molecules without the need of their physical existence^13^. Such algorithmic approaches have become an indispensable armamentarium for discovering novel drugs. There are several success stories, against GPCRs^29,30^ of identification of novel molecules by virtual screening. We were inspired by the aforementioned strategies and therefore screened small molecules for array of immune receptors, which play pivotal role during morbid pathological conditions. Furthermore, the available immunomodulatory therapies targeting the immune receptors include fusion and recombinant proteins, monoclonal antibodies, adjuvants and immune conjugates, vaccines, and gene therapies^31^. Majority of such biologics targeting immune receptors are more complex than small molecules or generic drugs. These involve complex production facilities and high cost of manufacture, shorter shelf life and specialized storage requirements. This inevitably results in variable immunogenicity and efficacy that may be attributed to product formulation process and host related factors^32–36^.

As a part of the present study, we are preparing central repertoire; ImmtorLig_DB using online SBVS pipeline software to screen a collection of small molecules for an array of receptors that are expressed by the cells of the immune system and play a cardinal role in bolstering the immune system against pathogens. As indicated in literature^13,37,38^, we addressed the major requirements for a successful SBVS to ensure quality and quantity of the screened small molecules. In the final part of study, we have utilized clustering and binning approach to determine the structural relatedness of the molecules that bind to a particular immune receptor and estimated ADME properties of each screened small molecule. ImmtorLig_DB with 5000 screened small molecules against an array of immune receptors can help a community of researchers with little or no background of chemoinformatics in exploring potential novel ligands to target immune receptors. Consequently, it will expedite the process of drug discovery.

## Methodology

### Establishing 3D structure of the immune receptor

Success of SBVS depends upon quality of the target structure^13^ and literature studies supported use of crystal structures with resolution less than 3.5 Å, as an optimum criteria for algorithms like SBVS^38,39^. In the present study, high resolution X-ray structures having resolution less than 3.5 Å were either obtained for virtual screening or were used as a template for homology modeling from Protein Data Bank (PDB). The databank was screened to shortlist the available crystal structures of the following immune receptors of human: (i) innate receptors: toll like receptors (TLR-1/TLR-2, TLR-4/MD-2, TLR-2/TLR-6), mincle; (ii) major histocompatibility complex 1 and 2 (MHC-I, MHC-II); (iii) co-stimulatory molecules CD28, CD40, CD80, CD86; (iv) co-inhibitory molecules: CTLA-4, PD-L1, Tim-3, decoy receptor, Fas ligand, Fas receptor; iv) cytokines: IL-1β, IL-2, IL-4, IL-6, IL-17, IL-23; (v) adhesion molecules: ICAM, VCAM, CEACAM1. Further, templates were selected from available crystal structures in PDB to perform homology modeling for predicting 3D structure of immune receptors, whose crystal structures did not meet the above criteria. Multi-chain modeling approach was employed for deducing the heterodimer structures and monomeric forms. For this, online server, HOMCOS 1.0 (http://homcos.pdbj.org/cgi-bin/index.cgi?LANG=en)^40^ was employed to screen and list the similar PDB templates and then modeling was performed employing Modeller version 9.17^41^. Protein homology modeling web server CPHmodels 3.2 (http://www.cbs.dtu.dk/services/CPHmodels/)^42^ was used to predict structure for some immune receptors. Such receptors whose structures were predicted prior to screening of ligands were highlighted in the database. In addition, receptor preparation involving protonation of amino acids, optimizing hydrogen bonds, assigning partial charges, relieving steric clashes in the structure were performed prior to virtual screening steps^13^. Structures were visualized using PyMol (http://www.pymol.org).

### Delineating or predicting ligand binding site for immune receptors

Ligand binding sites typically have concave cavity on the receptor molecule that has appropriate amino acid residues, where potential drug may bind^43^. Such sites can be the active sites of an enzyme, where receptor is formed with other moieties or the essential amino acid residues motifs that are necessary for functioning of the receptor^13,43^. The present study used the information obtained from co-crystallized structures of bound biological or synthetic ligand of the immune receptors in identifying structure motifs that are important for carrying out physiological function. This was achieved by using PyMol and highlighting and listing the interacting amino acid residues within the 4 Å region of the co-crystallized ligand. In past, such an approach in designing novel ligands have been successfully employed for GPCRs^44^. *In silico* prediction of ligand binding sites was carried out for immune receptors that were not co-crystallized with biological or synthetic ligand, using PocketPickker (CLIPPERS) feature available in Blaster Dock version 1.6.0^45^ or CASTp^46^ (Computed Atlas of Surface Topography of proteins) server (http://sts.bioe.uic.edu/castp/).

### Identification of small molecules for the target receptor by DOCK Blaster

An important prerequisite for a virtual screening algorithm is its ability to access a database of drug like compounds. The cataloging and number of entries in such databases are major benchmarks that dictate the performance and subsequent outcome of VS algorithms, both in terms of quality and quantity^10,37^. Screening process was initiated by docking over 30 million small molecules from ZINC database on a specified flexible ligand binding sites of respective targets. The small molecule sampling scheme has been set to “finer” that uses 55 “hots spots” and narrower bins along with “normal” scoring scheme utilizing standard AMBER 94 partial atomic charges on protein. The ranking score for each conformation is a combination of Van der waals forces (Vdw) and Electrostatic interaction (ES), corrected by partial ligand desolvation (Desolv) to obtain the free energy of binding (∆G). Based on the values of ∆G, top 200 small molecules were identified for each immune target and these were subjected to 100 steps simplex rigid body minimization^47^.

The above stated screening process was performed using the automated interface of DOCK Blaster version 1.6.0 (http://blaster.docking.org/)^47^ that has a pipeline of six modules, namely parser, scrutinizer, preparer, calibrator, docker, and assessor modules. These include identifying the receptor, resolving disorders, protonation and scoring grid assignment, computing scoring parameters, screening specified ZINC database subset over multiple CPUs, and preparation of results.

### Clustering analysis of array of small molecules that binds to immune receptors

Small molecules screened for each immune receptor were clustered in bins and then distributed over a scatter plot. The intent was to establish the structural relatedness of the molecules by finding a common molecular scaffold across the screened small molecules using ChemMine Tool (http://chemmine.ucr.edu/)^48^. The simplified molecular-input line-entry system (SMILES) for each small molecule was imported into the workbench of ChemMine Tool followed by binning into the cluster and 3D multidimensional scaling (MDS). The cutoff similarity score was defined at 0.4 (Tanimoto coefficient) for both of these.

### Establishing basic ADME properties for screened small molecules

Computational approaches are among the fastest methods for evaluating efficacy of the potential drugs^49^. SwissADME (http://www.swissadme.ch) computed ADME properties of the small molecules, by providing SMILES as the input. Following ADME parameters were computed: (i) physicochemical properties *viz*; molecular weight, number of heavy atoms, aromatic heavy atoms, fraction Csp3, rotatable bonds, hydrogen bond acceptors, hydrogen bond donor, molar refractivity, and Topological Polar Surface Area (TPSA); (ii) lipophilicity is computed using five different predictive models based on partition coefficient between n-octanol and water (log Po/w). A consensus log Po/w computed using arithmetic mean of the five models is also provided; (iii) water solubility is computed using three different models *viz*; ESOL, Ali, and SILICOS-IT; (iv) pharmacokinetics parameters such as GI absorption, BBB permeability, Pgp (permeability glycoprotein) substrate, inhibitor of five isoforms of cytochromes P450 (CYP) [CYP1A2, CYP2C19, CYP2C9, CYP2D6, CYP3A4], and skin permeation; (v) drug-likeness is predicted using filters like Lipinski, Ghose, Veber, Egan, Muegge, and bioavailability score calculation; (vi) medicinal chemistry, including structural alerts like PAINS and Brenk alert, leadlikeness, and synthetic accessibility analysis of the ligands^49^.

### Cataloging top 200 small molecules for each target protein into a database

The collected data of virtually screened small molecules were ranked according to their binding affinity, empirically measured in terms of free energy of binding (∆G) against the immune receptors. Further, for each small molecule screened, availability information, clustering analysis and the predicted ADME properties were accordingly cataloged in the form of a searchable database. The Entity Relationship (ER) diagram, depicting the flow of information among fields of ImmtorLig_DB was also created.

### Structural comparison of virtually screened small molecules

In silico screen ligands of TLR-4/MD-2 and ICAM were subjected to structural molecular similarity comparison with the experimentally tested ligands of the same receptors. A list of these tested ligands was retrieved from PubChem^50^. This structural comparison of screened small molecules was performed using the text based biophysical properties and intermolecular similarities comparison protocol proposed by Vidal *et al*.,^51^. Also, all the small molecules screened were compared among themselves based on values of Tanimoto coefficient. The coefficient values were in the range from 0 to 1, with 1 depicting complete identity and 0 indicating no similarity.

## Results

### Selection of high resolution structure of immune receptors

**Figure 1:**
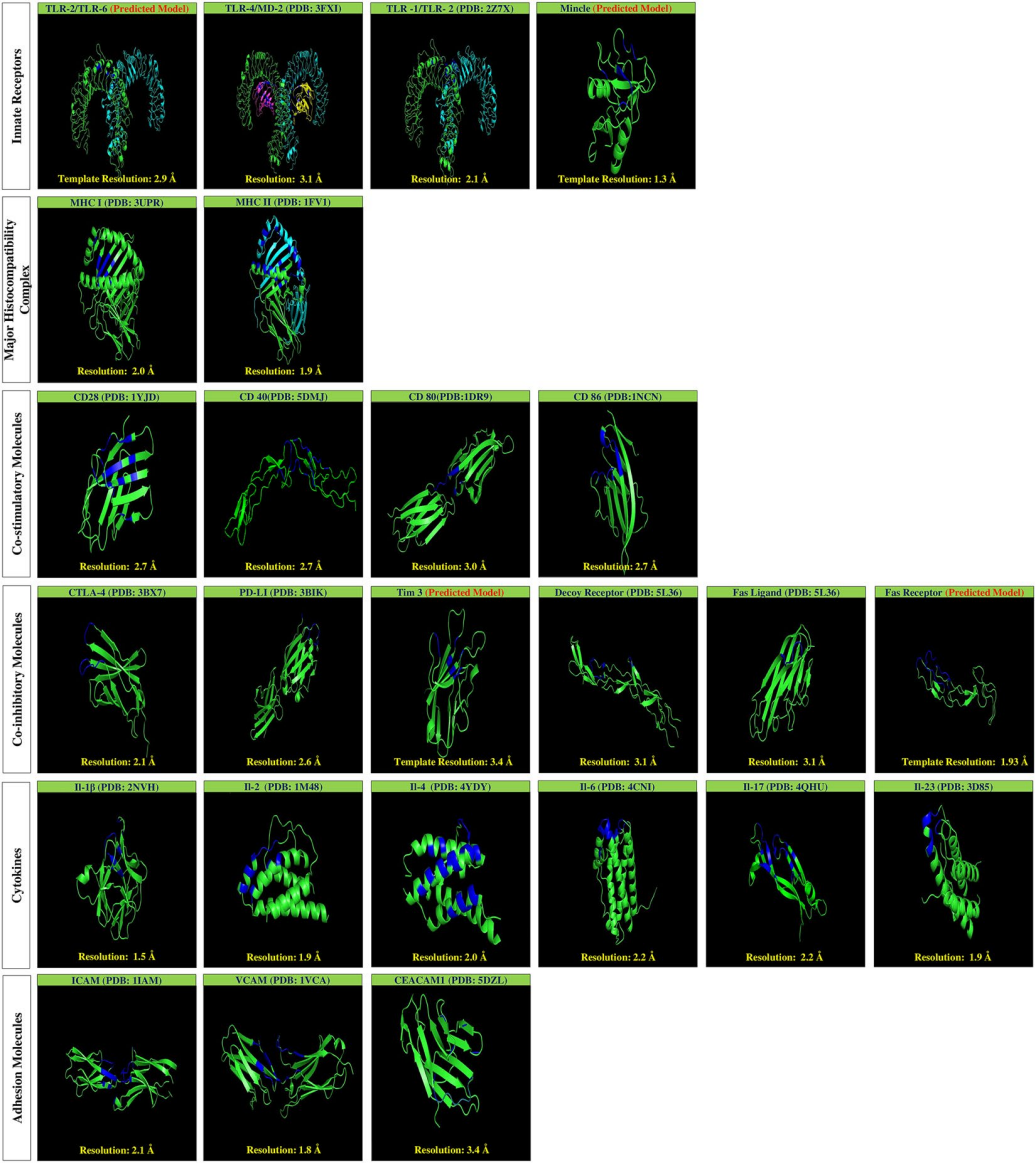
High resolution crystal structures and homology models of the immune receptors. 3D structures of following immune receptors; (i) innate receptors: toll like receptors (TLR-1/TLR-2, TLR-4/MD-2, TLR-2/TLR-6), mincle; (ii) major histocompatibility complex 1 and 2 (MHC-I, MHC-II); (iii) co-stimulatory molecules CD28, CD40, CD80, CD86; (iv) co-inhibitory molecules: CTLA-4, PD-L1, Tim-3, decoy receptor, Fas ligand, Fas receptor; iv) cytokines: IL-1β, IL-2, IL-4, IL-6, IL-17, IL-23; (v) adhesion molecules: ICAM, VCAM, CEACAM1. PDB code of the crystal structures and its resolution are appropriately indicated for the immune receptors. Blue colour are indicative of the ligand binding site, around which small molecules were screened. Crystal or the predicted structures were visualised using PyMol.

High resolution crystal structures of human immune receptors were obtained from PDB for the following: TLR-1/TLR-2, TLR-4/MD-2, MHC-I, MHC-II, CD28, CD40, CD80, CD86, CTLA-4, PD-L1, decoy receptor, Fas ligand, IL-1β, IL-2, IL-4, IL-6, IL-17, IL-23, ICAM, VCAM and CEACAM1. Further, templates from PDBIDs- 3A79, 3WH2, 5DZL, and 3TJE were selected for performing homology modeling of immune receptors *viz*; TLR-2/TLR-6, mincle, Tim-3, and Fas receptor. A template of PDBID 3A79 was used for modeling the heterodimeric structure of TLR-2/TLR-6, employing a crystal structure of *mus musculus* TLR-2/TLR-6. Homology modeling using Modeller version 9.17 was used to predict the heterodimeric structure of TLR-2/TLR-6, and monomeric form of Fas receptor. Similarly, CPH models server was used for modeling the structure of mincle and Tim-3. The crystal or predicted structures, along with their resolution for the immune receptor are presented in Fig. 1.

**Figure 2:**
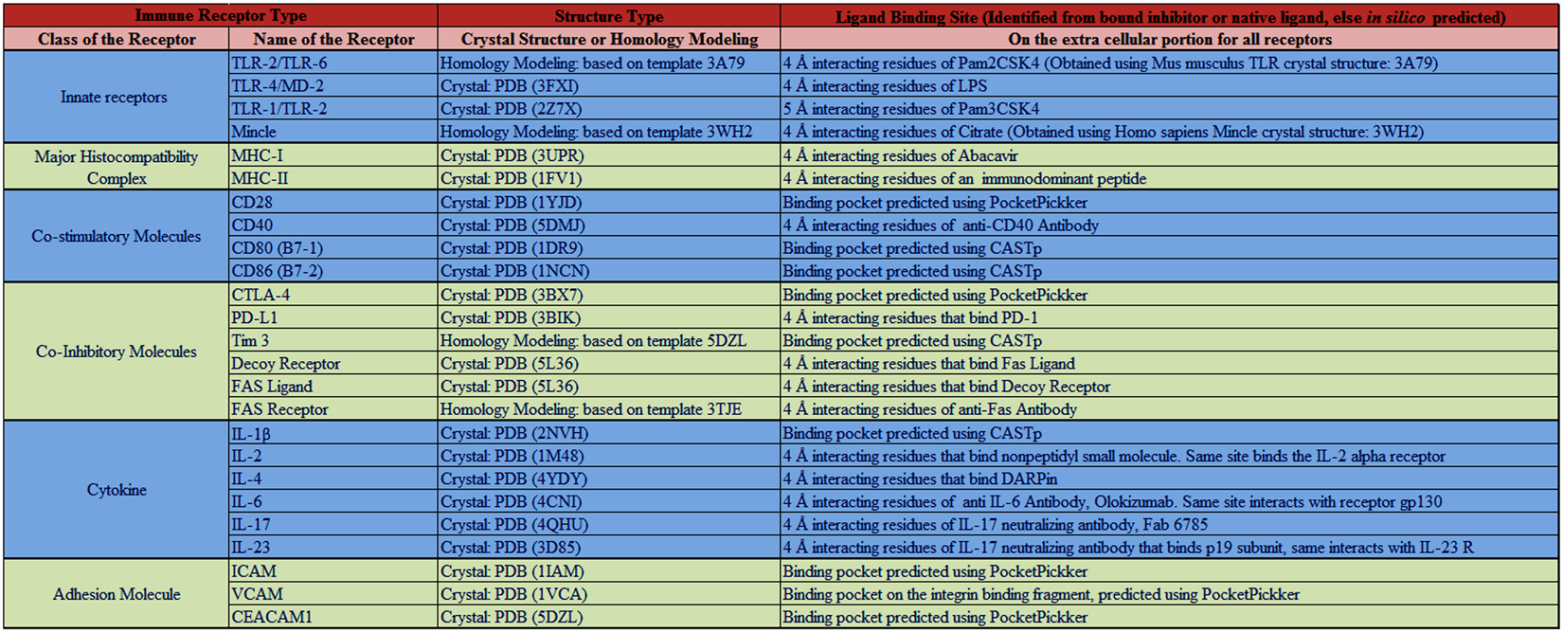
Essential domains of immune receptors considered as ligand binding site. PDB code of crystal structure or template used in modeling immune receptors are accordingly mentioned. The ligand binding sites were defined by the residues located within 4 Å of any atom of ligand in the co-crystal structures. *In silico* prediction server, PocketPickker (CLIPPERS) or CASTp were employed to predict the largest binding groove on the immune receptors that were not co-crystallized with any ligand.

Further, following PDB crystal structures 2Z7X, 3FXI, 4ZRV, 3UPR, 1FV1, 5DMJ, 3BIK, 5L36, 1M48, 4YDY, 4CNI, 4QHU, and 3D85 were available as co-crystals with their respective biological or synthetic ligand. This enabled identifying putative ligand binding sites in the immune receptors *viz*; TLR-1/TLR-2, TLR-4/MD2, MHC-I, MHC-II, CD40, PD-LI, decoy, Fas ligand, IL-2, IL-4, IL-6, IL-17, and IL-23. Also, the structures of templates with PDB IDs, 3A79, 3WH2, and 3TJE used for modeling TLR-2/TLR-6, mincle, and Fas receptor were available in complex with their biological or synthetic ligands, assisting in identifying the amino acid residues in the ligand binding cavity (Fig. 2).

### Virtual screening for small molecules was performed on essential binding motifs of the receptors

The ligand binding sites for selected immune receptors were directly obtained from the co-crystallized structure of biological or synthetic ligands and their receptors. In addition, services of *in silico* servers, PocketPickker (CLIPPERS) or CASTp were utilized to identify the largest binding pocket present on the surface of immune receptors in case information about the ligand binding site could not be acquired from the available crystal structures. PocketPickker (CLIPPERS) was utilized to identify the largest binding groove on PDB IDs- 1YJD, 3BX7, 1IAM, 1VCA, and 5DLZ that were of immune receptors CD28, CTLA-4, ICAM, VCAM, and CECAM, respectively. Similarly, CASTp was used for CD80, CD86 and IL-1β, having the PDB IDs- 1DRN, 1NCN, 2NVN respectively, in addition to identifying the binding groove of predicted structure of Tim-3. Figures 1 and 2 depicts PDB ID for specifying the immune receptors and also contains the information about ligand binding sites where virtually screened small molecules were docked. Amino acid residues in the ligand binding sites of the immune receptors are indicated in the Supplementary Table 1.

### Two hundred high affinity small molecules were screened for each immune receptor

**Figure 3:**
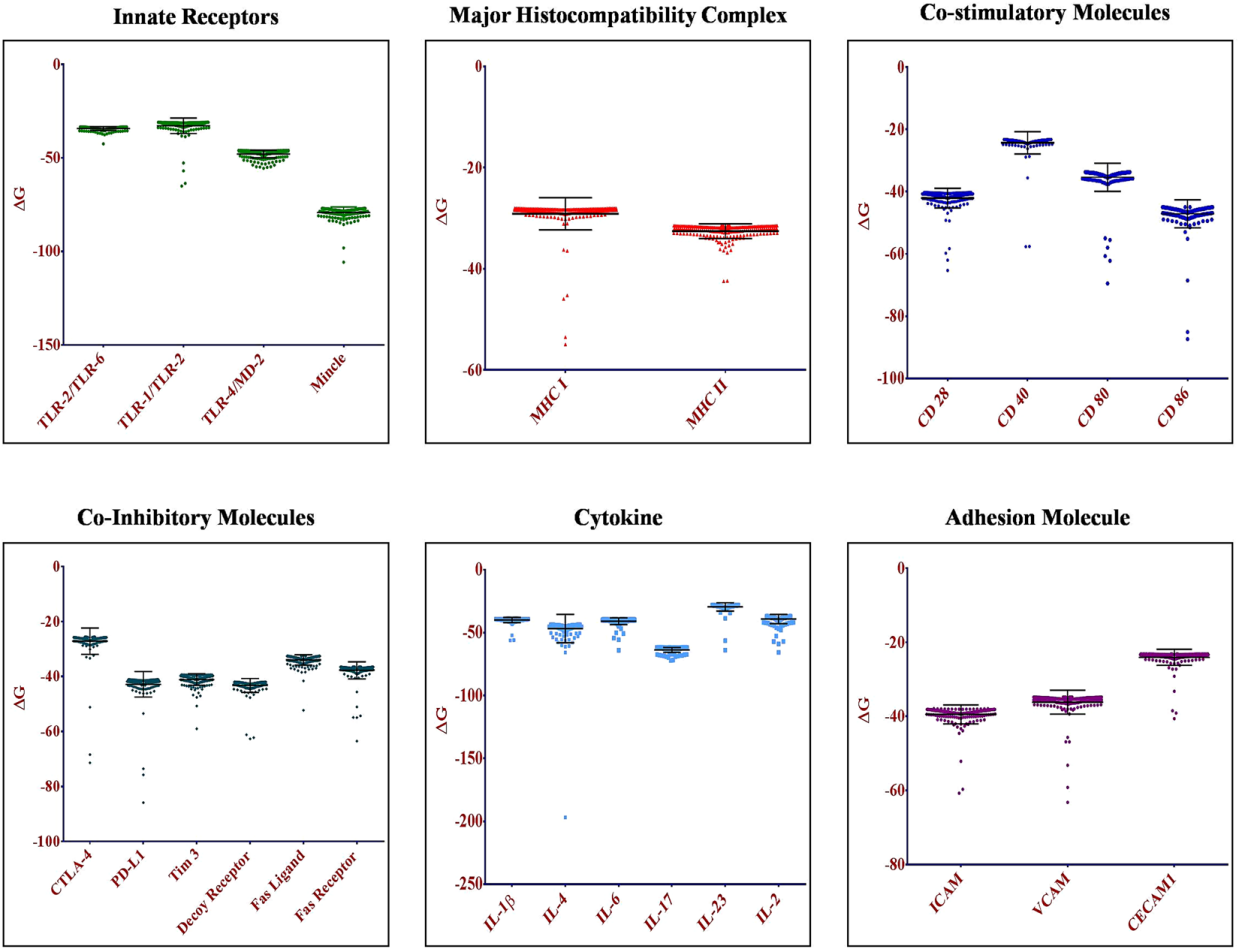
Small molecules that were screened exhibited strong efficacy for their representative receptors. Figure depicts average free energy of binding (ΔG value) along with the standard deviation for screened 200 small molecules against their respective immune receptors.

The screening of small molecules present in ZINC database was achieved by ranking the docking scores, obtained in terms of free energy of binding (∆G) after performing docking. Subsequently, average docking score of top 200 screened small molecules revealed high efficacy for selected immune receptors as compared to rest of the molecules (Fig. 3). The average free energy of binding, ∆G for the small molecules binding innate receptors *viz;* TLR-1/TLR-2, TLR-4/MD-2, TLR-2/TLR-6, and mincle were −32.822, −47.899, −34.309, and −79.193 kcal/mol, respectively. For first signal *viz*; MHC-I and MHC-II, the average ∆G for small molecules was −29.127 and −32.562 kcal/mol, respectively. For co-stimulatory molecules *viz*; CD28, CD40, CD80, and CD86, the average ∆G for the small molecules was −42.072, −24.312, −35.384, and −47.12 kcal/mol, respectively. For co-inhibitory molecules *viz;* CTLA-4, PD-L1, Tim-3, decoy receptor, Fas-L and Fas-R, average ∆G for the small molecules was −27.177, −42.854, −41.129, −43.255, −33.977, and −37.761 kcal/mol, respectively. For cytokines *viz*; IL-1β, IL-2, IL-4, IL-6, IL-17, and IL-23, average ∆G for the small molecules was −39.953, −39.198, −46.717, −40.907, −63.766, and −29.487 kcal/mol, respectively. For adhesion molecules *viz*; ICAM, VCAM, and CECAM, average ∆G of small molecules was −39.442, −36.147, and −24.054 kcal/mol, respectively. Thus, strong efficacies of the screened small molecules for the immune receptors are revealed by the negative value of average free energy of binding (∆G) obtained via docking process.

### Clustering analysis reveals structural resemblance among the small molecules binding an immune receptor

**Figure 4:**
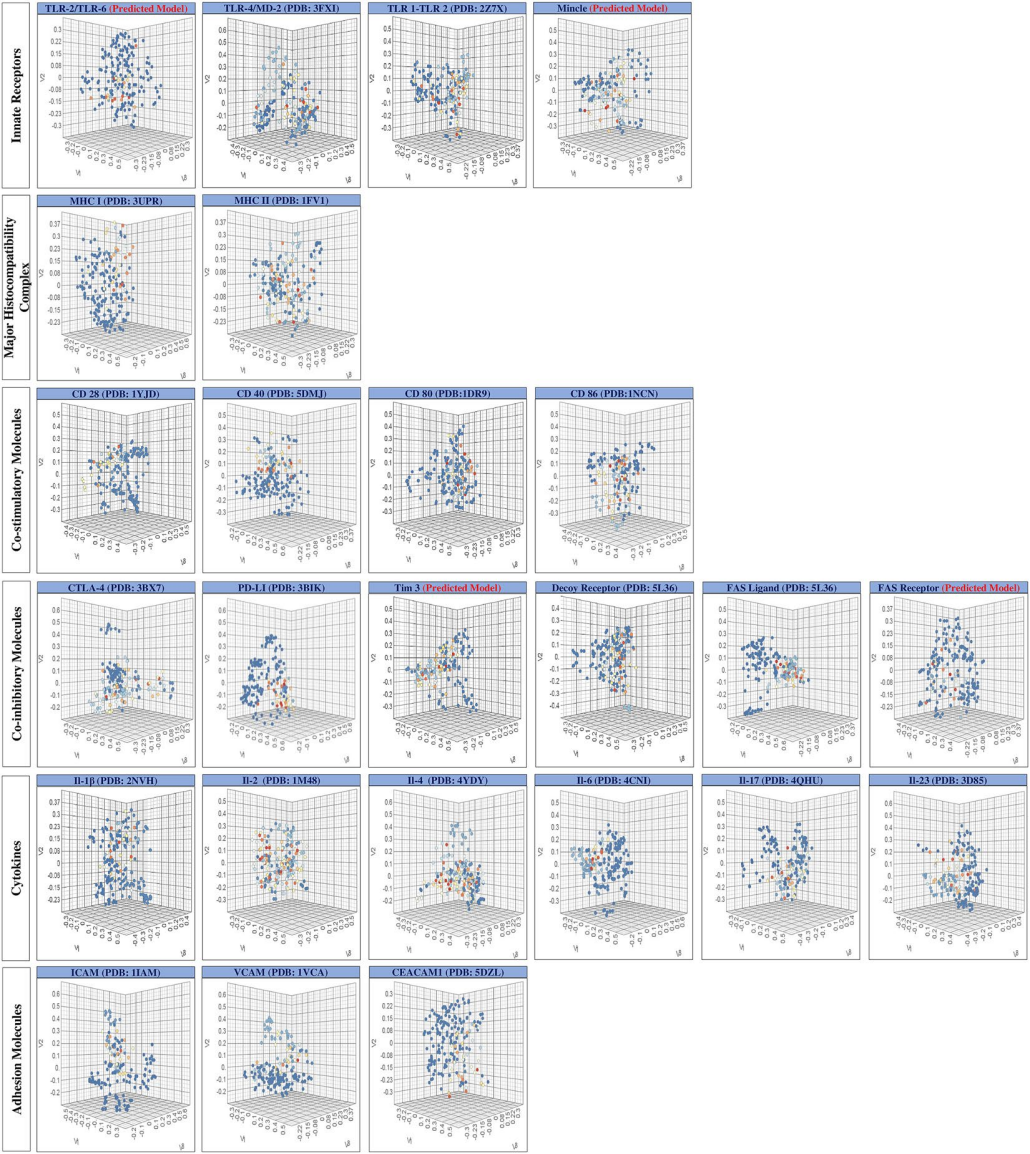
Multidimensional scaling suggests structural semblance. Clustering analysis was performed on the small molecules to obtain 3D scatter plot. Individual dots represent position of the ligands in a virtual space and distance among the dots reveals degree of relatedness. Confined dots in a form of dense bunches indicate the high degree of structural similarity among the screened small molecules.

**Figure 5:**
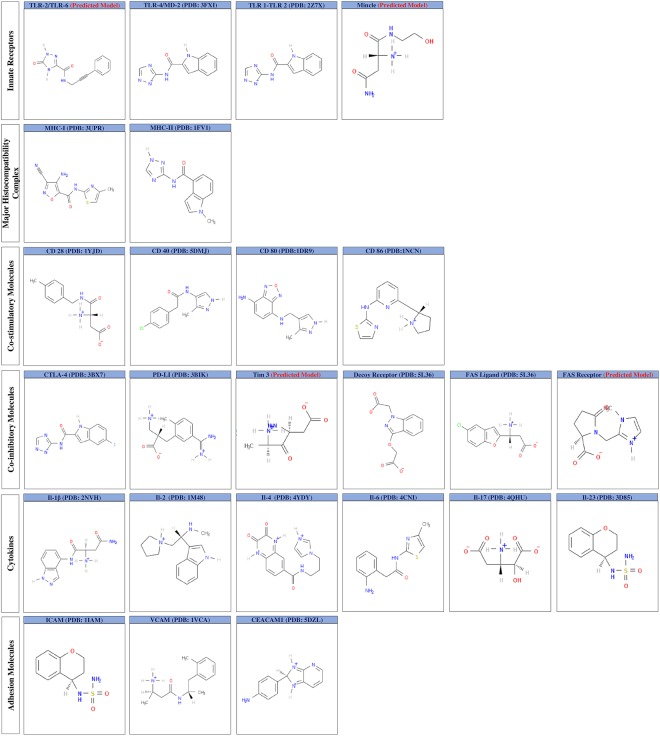
Representative structure of small molecules from largest bin for individual immune receptors. Binning performed with Tanimoto similarity coefficient cut off of 0.4 on the small molecules resulted in multiple bins having varied number of molecules. Representative small molecules from the largest bin for each of the 25 immune receptors are shown here.

Clustering analysis is a powerful tool for comparing chemical structures of molecules that reveals degree of structural similarities by identifying common molecular scaffolds^48^. Further, such common scaffold can be an indicative of bioactive profile of the molecule or its physicochemical properties, consequently correlating structural features with biological activities^48^. The present study performed Multi Dimensional Scaling (MDS) and clustering on the entire group of small molecules, binding to a particular immune receptor. The output obtained is in the form of a three dimensional scatter plot where individual dot represents a small molecule (Fig. 4). Each dot in the 3D scatter plot represents individual small molecule and the distance among dots reveals the degree of similarity among molecules. More packed bunches show more structural relatedness among the screened small molecules, and using the cursor of mouse, zinc ID of the corresponding small molecule can be viewed on the scatter plot in ImmtorLig_DB. Also, binning of cluster also helped to obtain the structural diversity among the collection of small molecules binding a particular immune receptor. We distributed the array of small molecules screened for an immune receptor into various similarity groups (or bins), with each bin having varied number of small molecules. All individual bins and the group of small molecules along with their zinc IDs for a particular immune receptor are available in ImmtorLig_DB. The representative structure from the largest bin, for each immune receptor is shown in Fig. 5.

### ADME properties reveal drug likeness of the small molecule binding immune receptor

**Figure 6:**
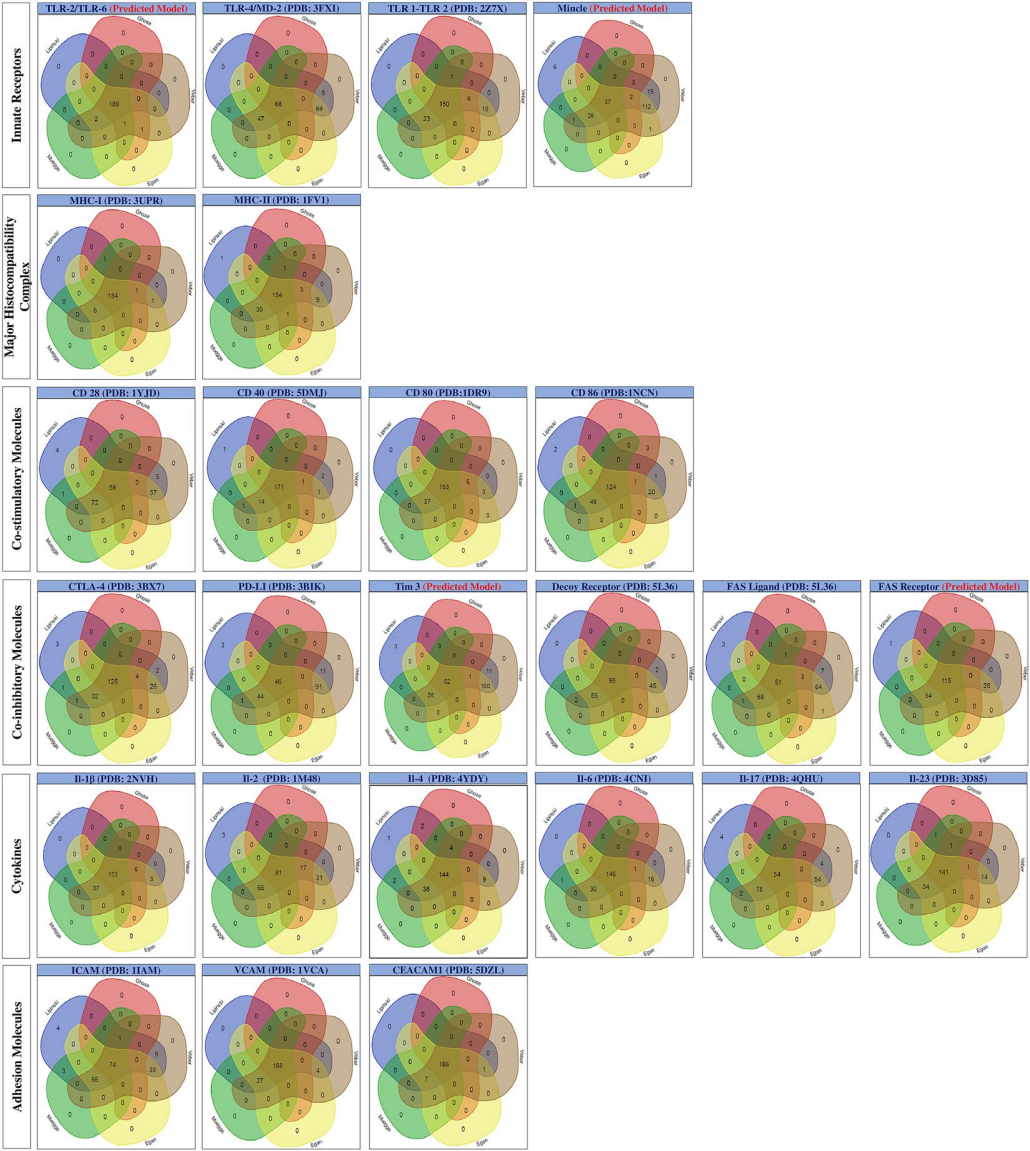
Five rule based filters reveals drug likness of the screened small molecules. Numbers of small molecules belonging to a particular set are shown in Venn diagram. Blue, red, brown, yellow, and green colour sets represent Lipinski, Ghose, Veber, Egan and Muegge drug likeness filter, respectively.

Estimation of ADME properties of potential drug leads is suggested prior to performing experimental assays, thus reducing the failure rates and saving of resources^49^. For 5000 screened small molecules, SwissADME enabled the prediction of major physiochemical properties, lipophilicity, water solubility, phyarmacokinetics, drug likeness, medicinal chemistry and the obtained results are presented in Supplementary Table 2. ImmtorLig_DB provides access to ADME properties for each ligand from database or separately view the entire list of properties for the group of ligands binding each type of the immune receptors. Majority of small molecule screened for each immune receptor were positive for all five drug likeness filter, as revealed by the maximum number of elements existing within the intersection of all 5 sets of filters (Fig. 6).

### Overall architecture of the ImmtorLig_DB

**Figure 7:**
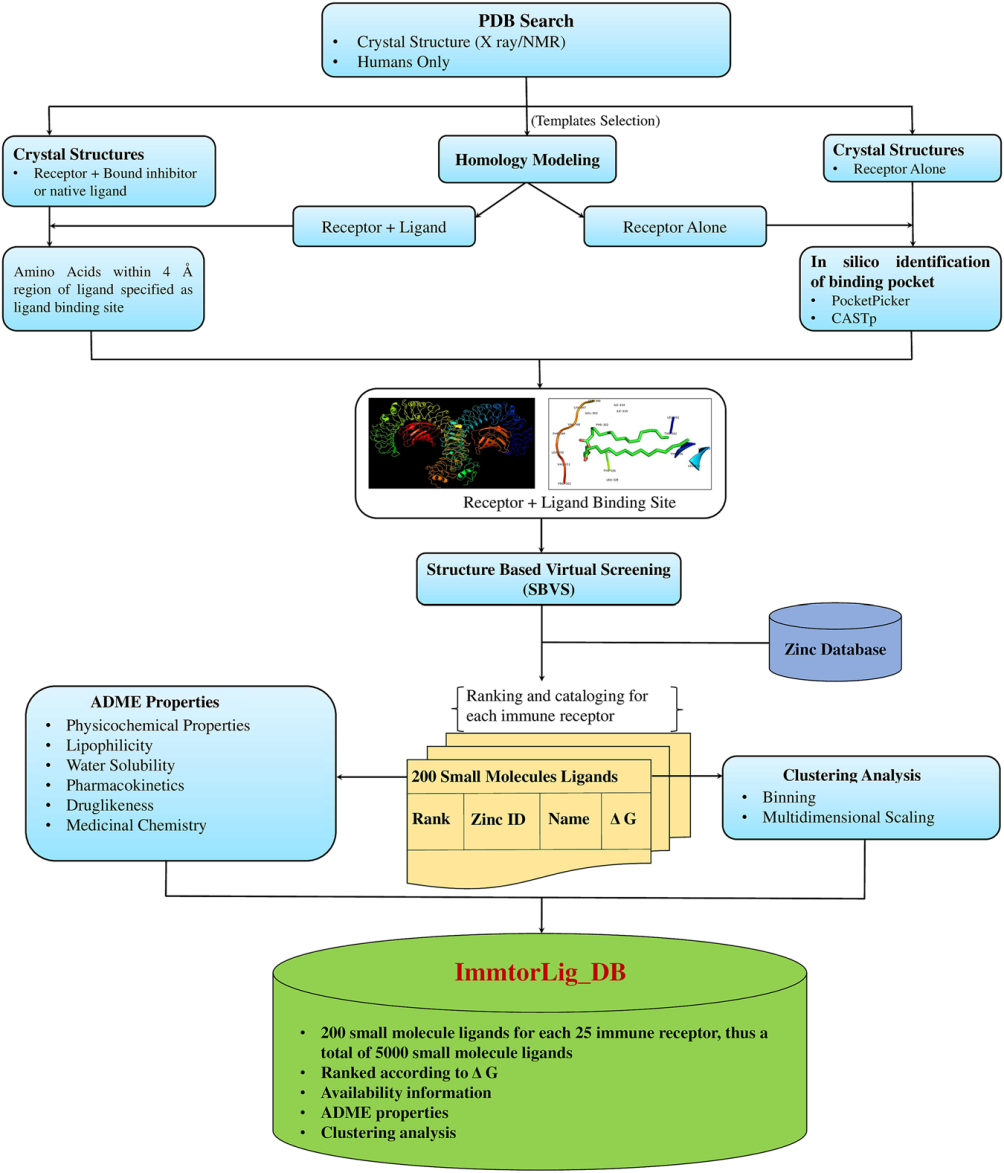
Overall architecture employed for ImmtorLig_DB. The graphical schematic representation of overall approach followed for collecting and analyzing the data of 5000 small molecules that were screened for 25 immune receptors.

The cataloguing and schema of ImmtorLig_DB is depicted in Fig. 7 and the entity relationship diagram is presented in Supplementary Fig. 1. The database was built on Apache HTTP server and MySql server 1.8.3-5. The front-end of ImmtorLig_DB was developed using HTML, PHP, and Javascript while MySql was engaged for back-end architecture. GUI based and searchable database is freely accessible through http://bioinfo.imtech.res.in/bvs/immtor/.

### Asserting the novelty of virtually screened ligands

**Figure 8:**
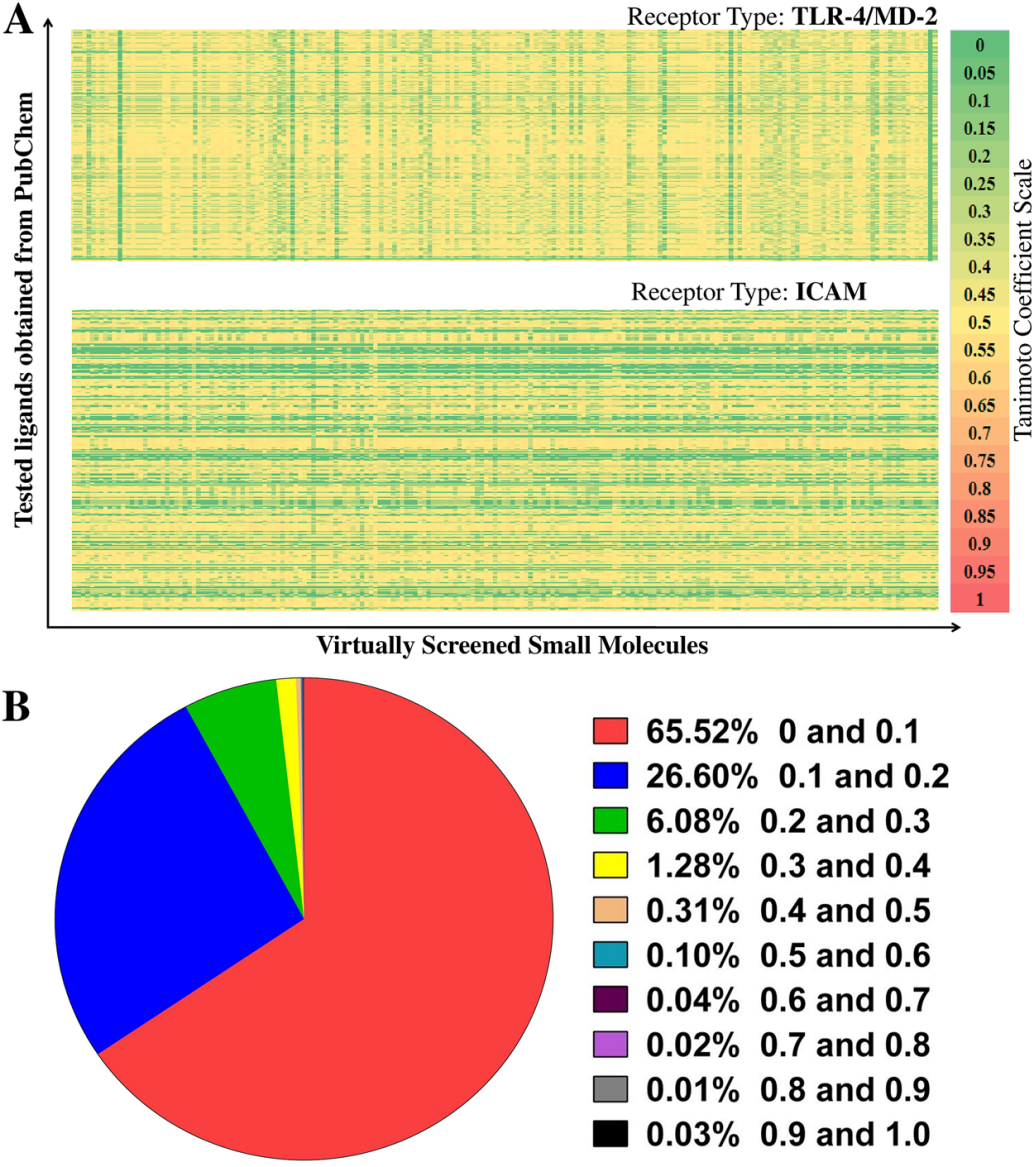
Structure based virtual screening indicates novelty for the small molecules. Based on the Tanimoto coefficient values, the structural relatedness among the screened small molecules were computed. The scale ranges from 0 to 1, where 0 signifies no similarity and 1 represents complete identity. (**A**) Heat map depicts the structural comparison between experimentally tested ligands available in PubChem and virtually screened small molecules for the immune receptors- TLR-4/MD-2 and ICAM. (**B**) Pie chart represents structural similarity among five thousand screened molecules, where 98.2% of small molecules had Tanimoto coefficient up to 0.3.

To explore the possibility that we obtained novel chemical structures among the virtually screened molecules, we have compared these with experimentally known and tested ligands of the receptors available in PubChem database^50^. Figure 8A is a heatmap representation of the degree of SMILES fragment based similarity between the prospective ligands obtained from virtual screening and the experimentally tested ligands from PubChem for the receptors TLR-4/MD-2 and ICAM. The color range in the heatmap is based on the Tanimoto coefficient values ranging from 0 to 1 calculated using the method of Vidal *et al*.,^51^. Thus, a value towards 1 or the orange/red color shade indicates near identity between SMILES string representations of two compounds while a value towards 0 or the yellow/green shade represents low similarity between the compared SMILES strings. Figure 8A shows a dominant occurrence of yellow/green shades indicating that a low similarity value range is observed for virtually screened compounds against PubChem compounds. Further, all virtually screened small molecules were structurally compared among themselves based on their Tanimoto coefficient and results of this comparison are presented in Fig. 8B. Majority of the small molecules i.e. 99.8% had Tanimoto coefficient values upto 0.5 and only 0.2% exhibited relatively high structural similar threshold of 0.5 to 1. In other words, the screened molecules themselves represent a structurally diverse repertoire. Thus, cataloged virtually screened small molecules in ImmtorLig_DB may be novel, and are structurally different from the experimentally tested ligands of the immune receptors.

## Discussion

Traditionally, GPCRs have been target for identifying small molecules using the combination of high throughput and virtual screening. Such approaches have been successful in identifying novel compounds or reduce the side effects of existing drugs by modifying the scaffolds^15–17,52^. Interestingly, computational approaches have been successfully applied in identifying novel molecules against immune receptors like TLR, ICAM, and cytokines^18–28,53^.

Immune receptors in the present study have been associated with many abnormalities, which range from autoimmunity, cancer, and developmental disorders. Major implications of identifying small molecules against the immune receptors of present study can be summarized in Table 1. The available therapeutic interventions targeting these immune receptors include immune adjuvant therapy, antibodies, and synthetic ligand based treatments. The demerits of such therapies include high manufacturing cost and specialized storage conditions, which make them difficult for mass production thereby leading to non availibility for the masses^32–36^. Small molecules have the ability to influence the activity of their receptor, thus, altering biological activity of the protein. Mechanism that explains this intrinsic change in the activity of a protein or receptor is the ability of the molecule to influence protein-protein interaction^54,55^

**Table 1:** Association of the immune receptors in context with the human diseases and list of available therapies in form of biologic drugs that target the immune receptors.

	Immune Receptor Type		Role in morbid conditions	Molecules targeting immune receptor
		Name of the receptor		
Class of the Receptor	Innate receptors	TLR-2/TLR-6	Innate receptor’s play an essential role in multiple diseases ranging from immune response during infection to establishing cancer immunity. Modulating activity of them is being used as therapy for such diseases.	PAM2CSK4, diacyl lipopeptides, lipoteichoic acid, zymosan, GPI anchor
		TLR-4/MD-2		LPS, mannan, glucuronoxylomannan, glycoinositolphospholipids, F protein of respiratory syncytial virus (RSV), envelope protein of mouse mammary tumor virus (MMTV)
		TLR-1/TLR-2		Pam3CSK4, triacyl lipopeptides
		Mincle		Trehalose-6,6-dibehenate (TDB), β-glucosylceramide
	Major Histo compatibility Complex	MHC-I	Malignant, infectious, autoimmune Disorders. Bare lymphocyte syndrome, transplant rejection	Maslimomab, abacavir, anti-CD8 antibodies
		MHC-II		Maslimomab, copaxone, cedelizumab, clenoliximab, ibalizumab, keliximab, priliximab, tregalizumab, zanolimumab, Ii-Key allosteric ligand
	Co-stimulatory Molecules	CD28	Autoimmune disease, atopic diseases	Theralizumab, CD28-SuperMAB, peptide-based antagonist
		CD40	Hyper-IgM immunodeficiency type 3, IBD, atherothrombosis, cardiovascular disease	Agonistic mAb (CP-870,893), teneliximab, monoclonal CD40L antibody
		CD80 (B7-1)	B-cell lymphoma, graft versus host disease, autoimmune diseases	Galiximab, abatacept, belatacept
		CD86 (B7-2)	Hodgkin’s lymphoma, thyroid carcinoma, allergic sensitization, acts as a receptor for adenovirus subgroup B	Anti CD86 blocking monoclonal Ab, abatacept, belatacept
	Co-Inhibitory Molecules	CTLA-4	Various forms of autoimmune syndromes, cancers and infectious diseases.	Ipilimumab, ticilimumab
		PD-L1	Different types of malignancies, infectious diseases	Nivolumab, pembrolizumab, pidilizumab, atezolizumab, avelumab, durvalumab
		Tim-3	Th1 mediated, and innate immune response during viral infection, exhaustion, cancer	Monoclonal antibodies (1G5, 2E2, and 4A4), anti-TIM-3 mAb (RMT3-23, ATIK2a)
		Decoy Receptor	Pleiotropic immunomodulator with role ranging from autoimmunity to cancer	Chemosensitization, recombinant TRAIL administration, conatumumab, mapatumumab, lexatumumab, tigatuzumab
		FAS ligand	Inducing apoptosis and killing of target cells, thus involved from lymphocyte homeostasis to autoimmune syndromes, graft-versus-host disease, and multiform of cancer.	Anti-Fas mAb *viz*; RK-8, h-HFE7A, gene therapy, synthetic peptide *viz*; residues 91-102 of DR5, TRAIL^mim/DR5^
		FAS receptor		
	Cytokines	IL-1β	Autoimmune syndromes, RA, pulmonary disease, diabetes	Canakinumab, rilonacept, anakinra, gevokizumab
		IL-2	Transplant rejection, MS	Daclizumab, basiliximab, cergutuzumab amunaleukin (CEA-IL2v), denileukin diftitox, gusperimus, tacrolimus, inolimomab
		IL-4	Asthma and inflammation	AMG317, pitrakinra, nuvance, AIR645
		IL-6	Types of arthritis and cancer, autoimmune diseases	Tocilizumab, sarilumab, siltuximab, clazakizumab, C326, olokizumab, elsilimomab
		IL-17	Autoimmune diseases, plaque psoriasis, ankylosing spondylitis, MS	Ixekizumab, secukinumab, brodalumab
		IL-23	Psoriasis and psoriatic arthritis	Ustekinumab, briakinumab, apilimod
	Adhesion Molecules	ICAM	Various types of cancer, renal disease, Alzheimer’s, IBD	Enlimomab pegol, ICAM-IONP
		VCAM	RA, development and inflammatory diseases	Natalizumab, vedolizumab, polyethyleneglycol modified immunoliposomes
		CEACAM1	Different types of malignancies	Anti-CEACAM6-maytansinoid (DM1), L-DOS47

. Therefore, there is an urgent need to identify small molecules that can be utilized as therapeutics, which are cheap and easy to manufacture in bulk and requires less stringent storage conditions.

Traditional approaches of identifying such novel small molecules from a chemical library, using expensive and laborious trial-error experimental assays have shown limited success^3^. Conversely with advancement in structural proteomics, *in silico* techniques, and high throughput algorithms, computer aided drug design has enabled tailoring of molecules against user specified target proteins. An approach like SBVS incorporate an armamentarium benefits that include^56–58^: i) time and cost reduction during the screening process, ii) a much larger chemical space can be virtually screened, thus significantly increasing the chances of finding novel small molecules, iii) moreover, such libraries need not be physically assessed as the initial approach does not depend on the experimental assays, iv) relatively simple in terms of the requirement of resources and manpower, iv) desirable ligands against a user specified target receptors can be screened, even if crystal structure of the receptor protein is not available. However, during the process of virtual screening of small molecules using algorithms, attrition rates and false positives are substantial challenges^59^.

Empirically, molecules binding a protein in the grooves which contain the catalytically important amino acids involved in carrying out the signal transduction process, have been implicated in altering the biological activity of that protein^60^. Therefore, the present study of virtual screening was performed on the ligand binding sites of the 25 immune receptors, thus creating a repertoire of 5000 small molecules that exhibits efficacious binding, and distinctive drug likeness.

Considering the novelity among virtually screened ligands, the mission of ImmtorLig_DB is to expedite the endeavour of scientists working in the field of immunology with the aim of discovering new drugs, as the therapeutic intervention for an assortment of morbid diseases. ImmtorLig_DB can serve as a ideal platform for understanding drug-target interactions that can be used in a compuational model to predict either small molecules or substructure fingerprint information of these. The present study, along with other widely used resources as discussed by Chen *et al*. can assist in developing such learning based computational models^61^ that shall employ neural network, support vector machines etc. for predicting small molecule and their putative immune targets. In addition to these techniques, random forest can also be used^62^ and further deep learning algorithms can make such methods more robust and effective for making predictions, as proposed by Wang *et al*.,^63^. This database is designed in a searchable and user friendly manner that will accelerate researchers across the globe with little or no prior knowledge or experience of virtual screening, chemoinformatics, or CADD in analyzing the lead compounds cataloged and to test the efficacy of these drugs. For this, the availability for each ligand mentioned in the database is to ensure a hassle free access of the ligands whose Zinc ID has been provided in the database.

## Conclusion

ImmtorLig_DB is a repository of 5000 small molecules binding to twenty five important immune receptors that seminally cortribute to morbidity and mortality of many human diseases. Two hundred small molecules have been virtually screened based on free energy of binding (∆G) for each of the immune targets. Majority of these small molecules are high affinity binders as revealed by their negative ∆G values. This repertoire includes potential novel chemical structures against immunological receptors as suggested by low structural similarity of virtually screened small molecules among themseleves as well as with experimentally tested ligands of TLR-4/MD-2 and ICAM. Clustering and binning analysis along with estimation of ADME properties of small molecules are additional features of this repertoire. In essence, ImmtorLig_DB can serve as an effective resource in the development of small molecule based therapeutic intervention for immune receptors.

## Electronic supplementary material

Supplementary Figure-1

Supplementary Table-1

Supplementary Table-2
