# Supplementary material for: ImmtorLig_DB: repertoire of virtually screened small molecules against immune receptors to bolster host immunity
Source: Sci Rep. 2019 Feb 28;9:3092. doi: 10.1038/s41598-018-36179-5 (PMC6395627; doi:10.1038/s41598-018-36179-5)
Supplement: Supplementary file 1 — Supplementary Figure-1 [file 41598_2018_36179_MOESM1_ESM.pdf]

ImmtorLig_DB: repertoire of virtually screened small molecules against immune receptors to bolster host immunity

Deepyan Chatterjee[1], Gurkirat Kaur[1], Shilpa Muradia[1], Balvinder Singh[1]*, Javed N Agrewala[1,2]*

[1]CSIR-Institute of Microbial Technology, Chandigarh-160036,

[1,2]Indian Institute of Technology Ropar, Rupnagar-140001, India

*Corresponding authors: Javed N. Agrewala, Indian Institute of Technology Ropar, Rupnagar, India

E-mail: jagrewala@iitrpr.ac.in, Balvinder Singh, CSIR-Institute of Microbial Technology, Chandigarh, India, E-mail: bvs@imtech.res.in.

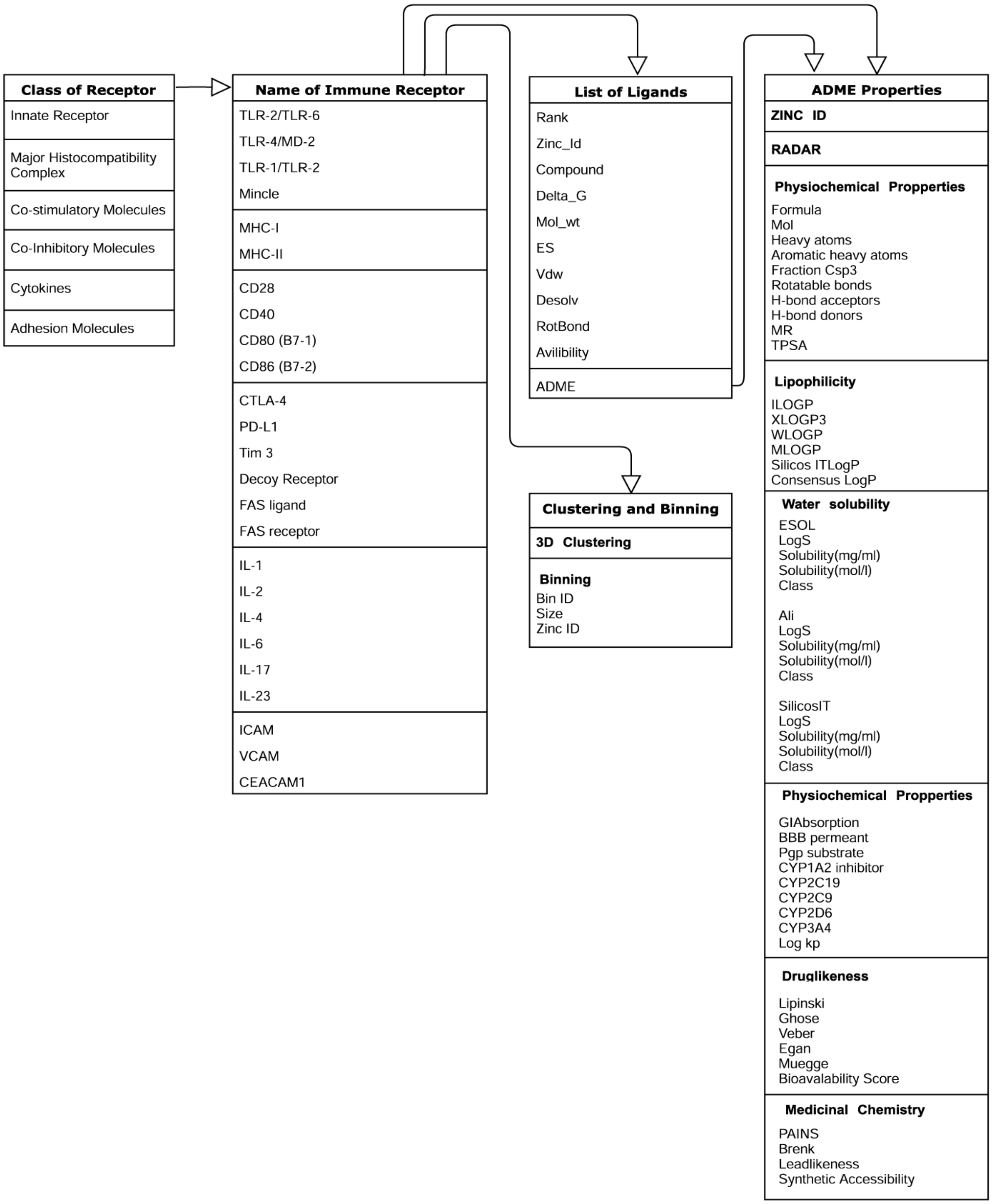

Supplementary Figure-1 Entity Relationship Diagram illustrates the flow of information and the relationship among the elements of ImmtorLig_DB.
